# Efficacy of pregabalin, amitriptyline, and gabapentin for neuropathic pain

**DOI:** 10.6026/973206300200386

**Published:** 2024-04-30

**Authors:** Prachi Dilipkumar Trivedi, Sarojini Posani, Neeharika Balla, Mohommed Mujtaba Sheezan, Ayesha Salim Hussain, Roshni Xavier, Kachhadia Meet Popatbhai, Mohammed Abdul Mateen, Priyadarshi Prajjwal, Mohammed Dheyaa Marsool Marsool

**Affiliations:** 1 GMERS Medical College, Himmatnagar, Gujarat , India; 2 Dr. Parveen Multi-speciality Clinic, Kothagudem, India; 3 Maharajah's Institute of Medical Sciences, Vizianagaram, India; 4 Medical University of Lodz , Poland; 5 Medical Officer, Ziva hospital, Kharar, Chandigarh; 6 Medical Officer at Carewell Hospital, Padapparamba, Malappuram, Kerala , India; 7 P.D.U Medical College, Civil hospital campus, Rajkot, Gujarat, India; 8 Shadan Institute of Medical Sciences Teaching hospital and Research Centre, Hyderabad, India; 9 Neurology, Bharati Vidyapeeth University Medical College, Pune, India; 10 University of Baghdad/Al-Kindy College of Medicine, Baghdad, Iraq

**Keywords:** Nerve pain, depression, anxiety, pregabalin, gabapentin, spinal cord injuries

## Abstract

Neuropathic pain largely influences the well-being of patients. Anticonvulsant and antidepressant medications, such as Pregabalin, Gabapentin, and Amitriptyline, are routinely prescribed as initial treatments for neuropathic pain. The study sample has a total of 270 patients who meet the inclusion criteria and are further distributed into three equally sized groups (A, B, and C). Group A was administered with Gabapentine 300mg, Group B with Pregabalin 75 mg, and Amitriptyline 10 mg to Group C. The occurrence of any adverse drug response was documented using the ADR reporting form, while the pain of the patient's post-medication was recorded using a numerical pain rating scale (NPRS). The comparison of the NPRS scores of all three groups "by using ANOVA test" both at baseline and after 15 days reveal that the differences between the three groups are statistically insignificant (p > 0.089). However, after one month of continuous use, the difference becomes slightly significant (I.e., p = 0.003). Gabapentin, pregabalin, and amitriptyline demonstrate similar effectiveness in alleviating neuropathic (NeP) pain. The study concludes that gabapentin is superior to both pregabalin and amitriptyline with fewer adverse effects, leading to improved patient adherence for long-term use.

## Background:

Neuropathic pain (NeP) arises from an injury or disease that disrupts the function of the somatosensory nervous system. [1] Post-herpetic neuralgia, Polyneuropathy, posttraumatic neuralgia, and surgical pain are examples of peripheral causes of NeP; however, spinal cord damage and stroke are the major core causes of NeP. [2] Serotonin and norepinephrine reuptake inhibitors (SNRIs), pregabalin, gabapentin, and tricyclic antidepressants (TCA) were all strongly recommended for application and suggestion as first-line treatment, according to recently updated Recommendations for NeP medication from the neuropathic pain special interest group are used globally. [3] A well-known analgesic and anticonvulsant drug is pregabalin. The Food and Drug Association (FDA) has authorized pregabalin as the first medication with a label for the therapy for neuropathic pain and postherpetic neuralgia. [4] Pregabalin is a successful therapy for neuropathic pain, as shown by preclinical and clinical trials. Studies on animals have aided in describing the processes behind its anti-hyperalgesia and antiallodynic effects. [5] Additionally, clinical research has demonstrated that pregabalin, either on its own or in conjunction with analgesics, helps treat pain and its accompanying indications, with the benefits dose-dependent. Due to its continuous efficacy, simple administration, and high tolerance among neuropathic pain sufferers, pregabalin offers several advantages. [6] Postherpetic neuralgia (PHN) is a frequent condition treated with gabapentin (GBP). GBP's affinity for calcium voltage-gated channels, which are present in the central and peripheral nervous systems, namely their alpha2-delta subunit, with a high affinity underlies its mode of action. This ability alters neurotransmitter release and lessens nerve cell excitability. [7] This method of action may have analgesic effects on persons who suffer from neuropathic pain. [8] Tricyclic antidepressant amitriptyline is frequently administered to manage persistent neuropathic pain. While the exact mechanism by which amitriptyline reduces neuropathic pain is still not fully understood, it inhibits the reuptake of noradrenaline and serotonin. [9] Unlike its action in treating depression, amitriptyline's analgesic effects are often achieved at lower dosages and side effects tend to diminish after a few weeks, revealing the drug's beneficial effects. [10] Additionally, there is no connection between how antidepressants affect pain and mood, nor do they analgesically affect both those with and without depression. [11] Therefore, it is of interest to assess the efficacy of pregabalin, amitriptyline, and gabapentin in the management of neuropathic pain.

## Materials and Methods:

The current research is a Prospective, Cohort, Open-label, three-arm study. The study was conducted between March 2022 and June 2023 by the Department of Medicine at Tertiary Care Teaching Hospital and the surrounding Primary Health Care centres.

## Inclusion Criteria:

The study sample included all patients who are at least 18 years old and diagnosed with low back discomfort associated with neuropathic pain, spinal cord damage, fibromyalgia, and post-herpetic neuroglia.

## Exclusion criteria:

History of diabetes mellitus, tuberculosis, heart, liver, or renal diseases or being pregnant/lactating at the time of research is used. Additionally, immunocompromised patients and those with a history of hypersensitivity to the study medicines are also excluded.

A total of 270 participants were enrolled in the study and randomized to receive the treatment. Gabapentine 300 mg was administered to Group A, Pregabalin 75 mg to Group B, and Amitriptyline 10 mg to Group C. The evaluation of pain was conducted at three different time points during the study: at the beginning (day 0), after 15 days, and after 30 days, using the NPRS (numeric pain rating scale). Additionally, the ADR reporting form was used to report any adverse medication reactions that patients reported or that clinicians saw during the research.

## Statistical Analysis:

In this study, we used three different statistical tests:

[1] ANOVA test: To contrast the mean pain as measured by the pain rating scale.

[2] Tukey Post Hoc test: to analyze and contrast the data between two groups at different time intervals.

[3] Chi-square test: To assess the negative medication effect across the three research groups.

For conducting all these statistical analyses, we used SPSS software version 20.

## Ethical:

The study was initiated after approval from the Institutional Ethics Committee for Medical Research at Shadan Institute of Medical Sciences, Teaching Hospital and Research Center. The IRB approval number is IRB/SIMS/03/25314/2022.

## Results:

Each group had 90 patients, with 37 (41.1%) males and 53 (58.9%) females comprising Group A. In Group B, there were 50 females (55.6%) and 40 males (44.4%). Group C comprised of a total of 34 males (37.8%) and 56 females (62.2%).

In our Group A, the patient age was 44.36 + 10.36 years. On the other hand, in group B, the average patient age was 45.53 + 9.53 years. The average patient age in group C was 46.53 + 9.55. Statistics showed that the F-value was insignificant at 0.344 and the p value was not. NPRS score for Group A was 10.48.54, for Group B 10.59.99, and for Group C, it was 10.38.32 (Table 2); however, these values were not statistically significant owing to an F-value of 0.849 and a p-value of 0.590. The Mean±SD of the NPRS score at 15 days was 9.292.64 for Group A, 9.494.05 for Group B and 9.994.15 for Group C. The F-value was 2.64, and the p-value was 0.089, which rendered the results statistically insignificant. With an F-value of 7.59 and a p-value of 0.003, the mean±SD of the NPRS score at one month was 6.444.75 in Group A, 6.804.91 in Group B, and 8.364.35 in Group C, suggesting statistical significance.

In the current study, group B had substantially higher cases of dizziness than Group A or Group C, with 25 patients (27.8% vs. 20 patients (22.2%) and nine patients (10%), respectively (p = 0.060). As opposed to group A's 30 patients (33.3%) and group C's 25 patients (27.8%), group B's 27 patients (30%) experienced sedation, a statistically significant variance (p=0.048). Nine patients in group C (10%) had constipation, substantially higher than in groups A and B (p=0.032). Compared to groups A and B, group C had 14 patients (15.6%) with dry mouth more frequently (p = 0.000).

## Discussion:

Neuralgia or neuropathic pain affects 7-10% of the population and is known to be highly persistent. [12]. It is characterized by abnormal activation of the nociceptive pathway caused due to various factors such as diabetes, multiple sclerosis, viral infections, and cancers [13]. Neuropathic pain significantly differs from the typical pain experienced by individuals [14]. The condition known as neuropathic pain is challenging to treat, and so far, there is no single effective treatment [15]. The first line of treatment usually involves antidepressants and anticonvulsants, particularly Amitriptyline, Pregabalin, and Gabapentine, which are currently used in the study. When the first line fails, lidocaine, botulinum toxin, and potent opioids can be used as second and third lines of treatment [16]. Many recent studies have discussed the three common drugs used in treating neuropathic pain "pregabalin, amitriptyline, and gabapentin" and concluded a set of recommendations based on randomized placebo-controlled studies [17]. However, only a small percentage of them have compared the drugs directly, "i.e., head-to-head comparison" [18]. This kind of comparison provides the best available data to compare the efficacy of the three therapies [19]. All three therapies were equally effective in decreasing pain; no statistically significant difference was consistent with earlier research conclusions. Our study's findings demonstrated that Amitriptyline and Gabapentine, Pregabalin, are comparable in relieving pain, and there was no discernible difference between the three therapies. Both drugs are beneficial in lowering neuropathic pain in earlier systematic reviews, which is consistent with the results of the current investigation. The efficacy of PGB and GBP were not contrasted as described elsewhere [20]. Numerous systematic reviews found no significant differences in the effectiveness of PGB, amitriptyline, and GBP in patients who reported a decrease in pain, which is statistically comparable to the outcomes of our investigation. However, data demonstrate that indirect comparisons are typically the outcomes of direct comparisons. [21] Compared to numerous earlier review studies that examined PGB and GBP with amitriptyline, the current study's conclusions differ. Based on those investigations, GBP is more effective than PGB at treating neuropathic pain.

## Conclusions:

Gabapentin, pregabalin, and amitriptyline demonstrate similar effectiveness in alleviating neuropathic (NeP) pain. In terms of NPRS score, gabapentin is superior to both pregabalin and amitriptyline. Gabapentin has been reported to have fewer adverse effects, leading to improved patient adherence for long-term use. However, amitriptyline offers a more economical alternative to pregabalin for further consideration.

## Limitations of the study:

The data with small sample numbers and the subpar methodological quality of the head-to-head investigations can be considered a drawback as it impeded thorough analyses of the results of the earlier research.

## Figures and Tables

**Table 1:** Overview of Patients' Gender and Age Demographics.

**Categories**	**Group A**	**Group B**	**Group C**
**Gender**			
Male	37 (41.1%)	40 (44.4%)	34 (37.8%)
Female	53 (58.9%)	50 (55.6%)	56 (62.2%)
Age group*			
18-40	16 (18%)	13 (14%)	17 (19%)
41-60	38 (42%)	39 (43%)	33 (37%)
>61	36 (40%)	38 (42%)	40 (44%)
Mean ±SD	44.36 ±10.36	45.53 ±9.53	46.53 ±9.55
*F-value = 0.344 and P-value = 0.636

**Table 2:** All three groups' NPRS scores were compared using an ANOVA at baseline, after one day and after 15 days.

**Duration**	**Mean ±SD**	**F value**	**P value**
**Baseline**			
Group A	10.48 ±2.54	0.849	0.59
Group B	10.59 ±2.99		
Group C	10.38 ±2.32		
After 15 days			
Group A	9.29 ±2.64	2.64	0.089
Group B	9.49 ±4.05		
Group C	9.99 ±4.15		
After 1-month			
Group A	6.44 ±4.75	7.59	0.003
Group B	6.80 ±4.91		
Group C	8.36 ±4.35		

**Table 3:** Comparison of the two groups' NPRS scores at the beginning, after one month, and after 15 days (Tukey post hoc test)

**Duration**	**Groups compared**	**Mean**	**P value**
Baseline	A versus B	0.26	0.64
	B versus C	0.24	0.73
	C versus C	0.25	0.744
After 15 days	A versus B	0.93	0.444
	B versus C	0.99	0.064
	C versus C	0.43	0.429
After 1-month	A versus B	0.16	0.46
	B versus C	2.6	0.015
	C versus C	2.75	0.009

**Table 4:** ADR in each of the three categories of individuals

**Symptoms**	**Group A**		**Group B**		**Group C**		**Chi-square**	**P- value**
	**N**	**%**	**N**	**%**	**N**	**%**		
Dizziness	20	22.2	25	27.8	9	10	6.53	0.06
Sedation	30	33.3	27	30	25	27.8	8.74	0.048
Constipation	0	0	0	0	9	10	9.53	0.032
Dry mouth	0	0	0	0	14	15.6	15.9	0
